# Snow at Great Altitudes

**Published:** 1881-03

**Authors:** 


### SNOW AT GREAT ALTITUDES DOES NOT MELT.

The reason why snow at great elevations does not melt, but remains permanent, is owing to the fact that the heat received from the sun is thrown off into the stellar space so rapidly by radiation and reflection that the sun fails to raise the temperature of the snow to the melting point. The snow evaporates, but it does not melted. The summits of the Himalayas for instance, must receive more than ten times the amount of heat necessary to melt all the snow that falls on them, notwithstanding which the snow is not melted. And in spite of the strength of the sun and the dryness of the air at those altitudes, evaporation is sufficient to remove the snow. At low elevations, where the snow-fall is probably greater, and the amount of heat even less than at the summits, the snow melts and disappears. This we must attribute to the influence of aqueous vapor. At high elevations the air is dry, and allows the heat radiated from the snow to pass into space; but at low elevations a very considerable portion of the heat radiated from the snow is absorbed in passing through the atmosphere. A considerable portion of the heat thus absorbed by the vapor is radiated back on the snow; but the heat thus radiated, being of the same quality as that which the snow itself radiates, is on this account absorbed by the snow. Little or none of it is reflected, like that received from the sun. The consequence is, that the heat thus absorbed accumulates in the snow till melting takes place. Were the aqueous vapor possessed by the atmosphere sufficiently diminished, perpetual snow would cover our globe down to the seashore. It is true that the air is warmer at the lower level than at the higher level, and by contact with the snow must tend to melt it more at the former than at the latter position; but we must remember that the air is warmer mainly in consequence of the influence of aqueous vapor, and that were the quantity of vapor reduced to the amount in question, the difference of temperature at the two positions would not be great.

---

A "DROP" is a variable quantity, although many people never think about this fact. The *Journal of Chemistry* says that the largest drop is formed by syrup of gum-arabic, forty-four to the dram, and the smallest by chloroform, 250 to the dram. As a general rule, tinctures, fluid extracts and essential oils yield a drop less than one-half the size of water, and acids and solutions give a drop but slightly smaller than water.

### A DOSE FOR THE DOCTOR.

Dr. X. is an eminent physician of Philadelphia, and, as is often the case with eminent physicians, is brusque and overbearing in manner. Among his office patients one morning was a gentleman who, after occupying exactly five minutes of the great man's time, took a ten-dollar note from his pocket, and inquired the amount of the fee.

"Fifty dollars," said the impatient medical man.

The patient demurred a little, whereupon the physician rudely remarked:

"Well, what do you expect to pay? Give me what you have got," and on receiving the ten-dollar bill, turned scornfully to his colored servant, and handing him the money, remarked:

"That is for you, Jim;" but lost his temper still more when his patient coolly said:

"I did not know before that you had a partner. Good-morning, doctor."

---

A New Orleans lawyer, in arguing a divorce suit, held that a husband had a legal right to make his wife stand in a corner with a clothes-pin on her nose. "If such mild means of compelling obedience are forbidden," he said, "what is to become of the husband's authority as the master of his household?"

---

Wasted Tenderness.—When so much of child sorrow and need is asking for love and pity, is it not melancholy that these holy affections are given to the dogs? The New York *Home Journal*, referring to the fashion of making pets of dogs, says: "The amount of luxurious tenderness that is bestowed on these worthless creatures by some of our wealthy citizens is so astonishing as to be almost unbelievable. It is not at all uncommon to see a carriage, with two men upon the box, driving through the park upon a pleasant morning with only a dog or perhaps a pair of them inside taking a sniff of fresh air. They have had their bath, their locks have been dressed, and if they are very shaggy dogs, such as poodles, Skye terriers, etc., fresh ribbons are in the braid or curl of their top-knots, and possibly a tiny cluster of fresh blossoms are tied in the same becoming fastenings. A sleek, short-haired dog is carefully blanketed, and perhaps stockinged, with crocheted comforts, the like of which only the fortunate few among children are able to possess."—*Pacific Rural Press.*

